# Intersubunit Concerted Cooperative and *cis*-Type Mechanisms Modulate Allosteric Gating in Two-Pore-Domain Potassium Channel TREK-2

**DOI:** 10.3389/fncel.2016.00127

**Published:** 2016-05-13

**Authors:** Ren-Gong Zhuo, Peng Peng, Xiao-Yan Liu, Hai-Tao Yan, Jiang-Ping Xu, Jian-Quan Zheng, Xiao-Li Wei, Xiao-Yun Ma

**Affiliations:** ^1^State Key Laboratory of Toxicology and Medical Countermeasures, Beijing Key Laboratory of Neuropsychopharmacology, Department of Biochemical Pharmacology, Beijing Institute of Pharmacology and ToxicologyBeijing, China; ^2^Anesthesia and Operation Center, PLA General HospitalBeijing, China; ^3^Department of Pharmacology, School of Pharmaceutical Sciences, Southern Medical UniversityGuangzhou, China

**Keywords:** TREK-2, two-pore domain potassium channel, 2-APB, allosteric regulation, intersubunit interaction, concatenated dimer

## Abstract

In response to diverse stimuli, two-pore-domain potassium channel TREK-2 regulates cellular excitability, and hence plays a key role in mediating neuropathic pain, mood disorders and ischemia through. Although more and more input modalities are found to achieve their modulations via acting on the channel, the potential role of subunit interaction in these modulations remains to be explored. In the current study, the deletion (lack of proximal C-terminus, ΔpCt) or point mutation (G312A) was introduced into TREK-2 subunits to limit K^+^ conductance and used to report subunit stoichiometry. The constructs were then combined with wild type (WT) subunit to produce concatenated dimers with defined composition, and the gating kinetics of these channels to 2-Aminoethoxydiphenyl borate (2-APB) and extracellular pH (pH_o_) were characterized. Our results show that combination of WT and ΔpCt/G312A subunits reserves similar gating properties to that of WT dimmers, suggesting that the WT subunit exerts dominant and positive effects on the mutated one, and thus the two subunits controls channel gating via a concerted cooperative manner. Further introduction of ΔpCt into the latter subunit of heterodimeric channel G312A-WT or G312A-G312A attenuated their sensitivity to 2-APB and pH_o_ alkalization, implicating that these signals were transduced by a *cis*-type mechanism. Together, our findings elucidate the mechanisms for how the two subunits control the pore gating of TREK-2, in which both intersubunit concerted cooperative and *cis*-type manners modulate the allosteric regulations induced by 2-APB and pH_o_ alkalization.

## Introduction

Two-pore domain K^+^ (K2P) channels, the last discovered K^+^ channel family, are major contributors to background K^+^ conductance by producing “leak” currents, and play a predominant role in stabilizing resting membrane potential as well as regulating cellular excitability. Up to now, 15 members, which are divided into six subgroups, have been found in mammals. TREK-2 (TWIK related K^+^ channel, K2P10.1), along with TREK-1 and TRAAK (TWIK-related arachidonic acid-stimulated K^+^ channel), belongs to the TREK/TRAAK K2P subfamily (Enyedi and Czirják, 2010). In addition to central nervous system (Lesage et al., 2000), the channel is also highly expressed in dorsal root ganglia (DRG) and trigeminal ganglia (Kang and Kim, 2006; Yamamoto et al., 2009; Acosta et al., 2014). In DRG C-fiber nociceptors, the functions of TREK-2 are involved in hyperpolarizing membrane potential and limiting spontaneous pain (Kang and Kim, 2006; Acosta et al., 2014). Evidence from knockout mice show the channel is associated with thermosensation and neuropathic pain (Pereira et al., 2014). Activating TREK-2 leads to GABAnergic and noradrenergic inhibition of neuronal excitability in the entorhinal cortex, which is regarded as the gateway to the hippocampus and thus is essential for learning and memory (Deng et al., 2009; Xiao et al., 2009). In response to ischemia, TREK-2 expression is up-regulated in the astrocytic membrane (Rivera-Pagán et al., 2015), cortical and hippocampal neurons (Li et al., 2005). Therefore, TREK-2 has been implicated to be a new potential therapeutic target for treating neuropathic pain, mood disorders and ischemia.

Similar with other K2Ps, single TREK-2 molecule is composed of four helical transmembrane domains (M1-M4) and two pore-forming domains (P-domain). Classical K^+^ channels need to tetramize to form the canonically four-fold pore, whereas dimerization is sufficient for K2Ps, due to their two P-domains are fused into one molecule subunit. Thus, the pore of K2Ps is assembled as a pseudotetrameric structure compared with classic K^+^ channels. There arranges an inner gate (the lower parts of M2 and M4) and a selectivity filter (SF) gate along the ion pathway (pore region) from outside to inside. As a thermo- and mechano-gated K^+^ channel, TREK-2 is also regulated by lysophospholipids, polyunsaturated fatty acids, G-protein coupled receptors, extra- or intracellular pH, and a range of clinically useful drugs including volatile anesthetics (Bang et al., 2000; Lesage et al., 2000; Deng et al., 2009; Sandoz et al., 2009; Xiao et al., 2009, 2014). It has been reported that some stimuli, such as Ba^2+^, can block K^+^ efflux by binding to the pore directly (Zhuo et al., 2015b). However, most of stimuli control the velocity or quality of K^+^ efflux primarily by coupling with the movements of pore region, a mechanism known as allosteric regulations. This mechanism is the most common and effective strategy to control protein activity, especially in allowing signal transmission over a long distance (Perutz, 1989). In multisubunit channels, intersubunit cooperativity might occur during conformational transition in the presence of stimuli or voltage gating. In voltage-dependent K^+^ channels (Kv), it has been established that voltage opens the pore via a concerted cooperative or sequential gating transitions in the tetrameric complex (Tytgat and Hess, 1992; Pathak et al., 2005; Zandany et al., 2008; Gagnon and Bezanilla, 2009, 2010; Yifrach et al., 2009; Meisel et al., 2012; Thomson et al., 2014; Wu et al., 2014a,b). However, no such progress is made in K2Ps. Although more and more signals have been found to act on TREK-2 channel to exert their biological functions, little is known about the putative mechanism of intersubunit interaction, and the manner of signal transduction along single subunit during gating.

Our previous studies have demonstrated that 2-Aminoethoxydiphenyl borate (2-APB), a membrane-permeable compound, stimulates the activity of TREK-2 via acting on its cytosolic proximal C-terminus (pCt; Zhuo et al., 2015a), and opens the pore regardless of the original conformation of SF gate (Zhuo et al., 2016). This allosteric transduction (named as “2-APB pathway”) is initiated from cytosolic Ct and propagated to SF gate. In addition, changes of extracelluar pH (ΔpH_o_) induce the conformational transition of SF gate in K2Ps via an external sensor (Clarke et al., 2008; Cohen et al., 2008; Stansfeld et al., 2008; Niemeyer et al., 2010; Ma et al., 2011; Zúñiga et al., 2011), including TREK-2 (Sandoz et al., 2009; Zhuo et al., 2015a). Such transition in TREK-2 is also facilitated by the pCt domain in TREK-2 (Zhuo et al., 2016). This transmission is evoked by the charge change of sensor and propagated such stimuli to SF gate, and ultimately to intracellular pCt domain, a process called as “pH_o_ pathway”. These two pathways (2-APB pathway and pH_o_ pathway) represent bidirectional and long-distance allosteric couplings between SF gate and pCt domain, and thus provide two facile models to study intersubunit interaction in TREK-2. Except for the pCt domain, the glycine hinge of M4 (G312) has been found to be another essential gating element, which controls the two pathways bidirectionally (Zhuo et al., 2016). Here, to determine the nature of signal transduction along single or double subunit, we constructed a concatenated dimeric TREK-2 channel and incorporated separately or simultaneously the two negative gating mutations, ΔpCt and G312A, into single or double subunits. We investigated the effects of these mutations or deletions in the context of tandem-linked dimers on both 2-APB pathway and pH_o_ pathways, and found that concerted cooperative intersubunit interaction and *cis*-type transduction along single subunit underlie the allosteric regulations of TREK-2 channels.

## Materials and Methods

### Molecular Biology

Human wild type (WT) forms of TREK-2 (NCBI reference sequence NM_138318)-expressing vector (pGH19-TREK-2) was constructed using *Bgl* II and *Hind* III sites. Point mutations and deletions were engineered using the MutanBEST kit (TaKaRa, Dalian, China)-guided high-fidelity PCR. The concatenated dimeric constructs were built to contain a different combination of WT and/or mutant (or deletant) TREK-2 cDNAs in defined position. The two consecutive subunits were connected by a flexible linker encoding the AAAGSGGSGGSTGGSSGSSGS sequence (Bagriantsev et al., 2012) with a little modification. A *Sal* I site was designed in the middle of the linker to facilitate cloning. One mutation (G312A) and two deletions (residues from W326 to A374 or from I323 to N543 were deleted in ΔpCt and ΔCt, respectively) were used in this study. G312 is located in the middle of M4 domain, and pCt is covalently linked with M4 and resides on the cytoplasmic side. For all the monomer (WT, G312A, ΔpCt and ΔCt) used to make dimers, *Bgl* II/*Sal* I was used to clone the former subunit, and *Sal* I/*Hind* III to the latter subunit. The name of concatenated dimers was defined as the relative position of monomeric channels. For example, WT-G312A represented that the first subunit was WT channel, and the second one was G312A channel. To investigate whether the flexible linker between monomers was degraded in mammalian cells during processing of the protein, either prior to or after membrane insertion, the concatenated WT dimeric construct (WT-WT) was also inserted into the pEGFPN1 vector (Clontech Laboratories, Mountain view, CA, USA) by using *Bgl* II and *Sal* I. All the constructs were confirmed by DNA sequencing. All the pGH19-based Plasmids were linearized by *X*ho I before *in vitro* transcription. cRNA was synthesized using the RiboMAX^TM^ Large Scale RNA Production Systems (Promega, Madison, WI, USA) kit.

### Channel Expression in *Xenopus* Oocytes

Procedures used for harvesting oocytes from *Xenopus laevis* were approved by the Animal Care and Use Committee at Beijing Institute of Pharmacology and Toxicology. After excised from *Xenopus laevis*, pieces of ovarian lobes were subjected to collagenase (Sigma Aldrich, St Louis, MO, USA) digestion. Stage V or VI oocytes were selected and cRNA (0.1~10 ng/46 nl for each oocyte) was microinjected. Injected cells were incubated at 18°C in ND96 medium (96 mM NaCl, 2 mM KCl, 1.8 mM CaCl_2_, 1 mM MgCl_2_, 10 mM HEPES, 5 mM pyruvate, 100 mg/ml gentamycin, pH 7.2).

### Electrophysiology

Whole-cell currents were measured 1–3 days after injection, and amplified using an Axoclamp2B amplifier (Axon Instruments, Union City, CA, USA) in two-electrode voltage clamp (TEVC) mode. Microelectrodes were pulled with a tip resistance of 0.1–1 MΩ when filled with 3 M KCl. Data were sampled at 2 kHz and filtered at 0.5 kHz with Clampex 10.0 software (Axon Instruments). Recordings were performed under standard, physiological extracellular solution (5 mM KCl, 93 mM NaCl, 1 mM MgCl_2_, 1.8 mM CaCl_2_, 5 mM HEPES, pH 7.4, adjusted with NaOH), and constant perfusion at room temperature. TREK-2 currents were elicited by continuous voltage-ramps from −120 to +60 mV from a holding potential of −80 mV, with 2 s in duration (current-voltage relationship, I-V curve). The currents of TREK-2 undergo obvious “run-up” at the beginning of recording, thus, each measurement was performed after the stabilization was reached (about 20 min) before applying stimulus. 2-APB (Promega, Madison, WI, USA) was diluted with the standard solution freshly.

### Cell Culture and Western Blotting Analysis

HEK 293 cells were cultured in DMEM supplemented with 10% fetal bovine serum and 2 mM L-glutamine and held at 37°C in humidified air with 5% CO_2_. Constructs encoding GFP (vector), TREK-2-GFP (monomer) and WT-WT-GFP (tandem-linked dimer) were transiently transfected into HEK 293 cells using Lipofectamine 2000 (Invitrogen, Carlsbad, CA, USA). Two days after transfection, the cells were harvested. Protein extracts were prepared by solubilizing in buffer (pH 7.4, 50 mM Tris, 270 mM NaCl, 1% Triton X-100) for 1 h and clarified by centrifugation at 12,000 × g for 30 min. Oocytes were injected with 10 ng cRNA, and then they were lyzed after 48 h in a cold buffer of 150 mM NaCl, 1.06 mM KH_2_PO4, 2.07 mM Na_2_HPO_4_, 1% Triton X-100, pH 7.4 supplemented with antiproteases and clarified by centrifugation at 20,000 × g for 15 min. The above lysates were subjected to SDS-PAGE on 10% gel and wet-transferred onto a PVDF membrane. The lysates were hybridized with anti-TREK-2 antibody (Abgent, San Diego, 1:200) or anti-GFP (Cell Signaling Technology, Danvers, MA, USA, 1:1000) for oocytes and HEK293 in Western blotting, respectively. Actin (anti-actin antibody was purchased from ZSGB-BIO, China, 1:5000) was used as loading control. Reaction bands were visualized by incubating the blots with appropriate fluorescence-tagged secondary antibodies (Lincoln, NE, USA, 1:15,000).

### Data Analysis

Voltage clamp data were analyzed with origin 8.0 (OriginLab Corporation, Northampton, MA, USA) and GraphPad prism version 5.0 (GraphPad Software Inc., La Jolla, CA, USA). The currents recorded at 0 mV were used to calculate all the ratios, which were presented as mean ± SEM, and reflected *n* = 5 or more from at least two batches of oocytes. Activation ratio (AR) was used to observe the stimulatory effects of externally applied 2-APB on TREK-2. AR was calculated from I_2-APB_/I_o_, where I_2-APB_ represented the currents in the presence of 2-APB, and I_o_ was the baseline, stabilized currents in the absence of 2-APB, and was plotted as function of the concentration of 2-APB ([2-APB]_o_). To estimate the inhibitory effects of extracellular alkalization, inhibition ratio (IR) was calculated from 1-I/I_pH6.5_, where I represented the currents in a specific pH, and I_pH6.5_ was the stabilized currents recorded at pH 6.5, and was plotted as a function of different pH (pH_o_). The resulted ratios were fitted to the Hill equation. Statistical differences were tested by using unpaired Student’s *t* test where appropriate. Differences were considered significant if *p* < 0.05.

## Results

### TREK-2 Concatenated Dimer Reserves Similar Gating Properties as its Monomer

**Figure 1:**
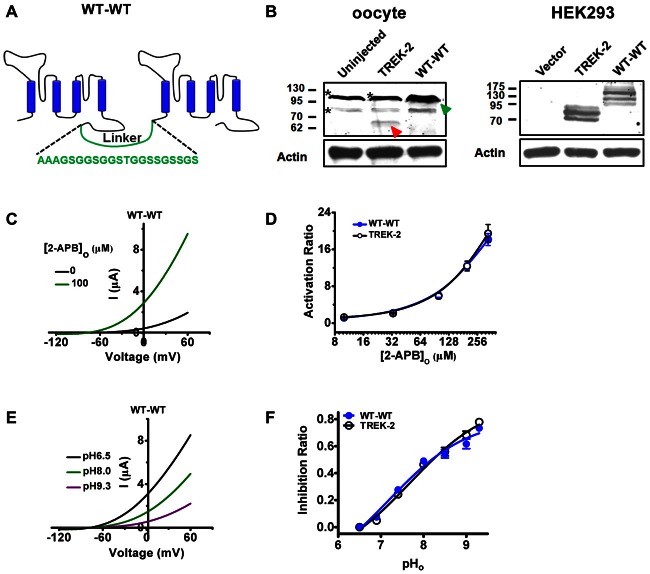
**Concatenated wild type (WT) TREK-2 dimers (WT-WT) functions similarly with their monomeric WT channels (TREK-2). (A)** Diagram of concatenated TREK-2 dimer and the sequence of linker indicated.** (B)** Validation of TREK-2 and WT-WT proteins was tested by western blotting. cDNA encoding TREK-2 or WT-WT was injected into oocytes. The monomeric TREK-2 was indicated with red arrow, and the dimeric one with green arrow. *Indicates the nonspecific bands detected by the anti-TREK-2 primary antibody in oocytes (left panel). Constructs harboring GFP-tagged TREK-2 or WT-WT was transfected into HEK293 cells (right panel). **(C)** Exemplar current-voltage recordings from oocytes expressing the WT-WT channels in the presence of 100 μM 2-APB. The currents were elicited by continuous voltage-ramps from −120 to +60 mV from holding potential of −80 mV, with 2 s in duration. **(D)** Comparative analysis of the 2-APB evoked activation curves between TREK-2 and WT-WT channels. **(E)** Exemplar current-voltage recordings from oocytes expressing WT-WT channels as pH_o_ transitions among 6.5, 8.0 and 9.3. **(F)** Concentration dependence of Inhibition ratio (IR) upon extracellular alkalization for TREK-2 and WT-WT channels.

Mature TREK-2 channel forms as homodimers, so mutations or deletions of the channel can involve two subunits simultaneously. To perform a quantitative study of intersubunit or intrasubunit interaction, a strategy that employs concatenated dimer was adopted. TREK-2 tandem constructs, in which the two subunits are connected by a 21-amino-acid flexible linker (Bagriantsev et al., 2012; WT-WT, Figure 1A) were produced and expressed in *Xenopus* oocytes. To investigate whether the tandem channels are properly expressed, we firstly detected the expressed proteins by monomeric (TREK-2) and concatenated dimeric constructs (WT-WT) in *Xenopus* oocytes using western blotting. As shown in Figure 1B (left panel), the monomeric construct produces one specific band corresponding to the approximate size of mature monomer (red arrow). Notably, two nonspecific bands were also detected by the anti-TREK-2 primary antibody in oocytes (asterisks). The WT-WT construct produces a specific band (green arrow, overlapped with a nonspecific band) corresponding with dimeric TREK-2. No monomeric signal was detected by In WT-WT construct-injected oocytes. To rule out the nonspecific expression, we further assessed their expressions in HEK293 cells by transfecting GFP-tagged monomeric and tandem-linked vectors. As shown in Figure 1B (right panel), distinct monomeric or dimeric bands were observed at the predicted molecular mass in TREK-2- or WT-WT-transfected group, respectively, but no monomeric signals were found in WT-WT group. Notably, no immunoreactivity was detected in the pEGFPN1-transfected cells (vector). These data indicate that the concatamers remain intact and the linker is not susceptible to degradation, and the WT-WT constructs can be used for further investigations.

To investigate the gating properties of the tandem-linked dimer, 2-APB response of the channel was investigated and compared with that of monomeric TREK-2. As the current-voltage relationships (I-V curves) shown in Figure 1C, the currents recorded from WT-WT channels was radically stimulated by 100 μM 2-APB. The activation ratio (AR, measured using the currents at 0 mV) values were plotted against their 2-APB concentrations, and results show that the activating effects of 2-APB on WT-WT channels was similar with that of monomeric TREK-2 (Figure 1D). The maximal AR (AR_max_, produced by 333 μM 2-APB, unless otherwise specified) produced by TREK-2 monomer and WT-WT tandem-linked dimer was 19.46 ± 1.94 and 18.13 ± 1.30, respectively (Table 1).

**Table 1:** **Response of TREK-2 tandem-linked channels and mutants to 2-APB and pH_o_ changes**.

	2-APB response		pH_o_ response	
Channel	AR^a^_max_	n^b^	IR^c^_max_	n^b^
WT-WT	18.13 ± 1.30	8–13	0.73 ± 0.02	8–9
ΔpCt-ΔpCt	4.34 ± 0.10	7	0.46 ± 0.01	7
WT-ΔpCt	15.50 ± 0.74	5–7	0.79 ± 0.01	5
ΔpCt-WT	17.37 ± 1.56	5–6	0.77 ± 0.02	6
WT-ΔCt	18.15 ± 2.42	5–7	0.76 ± 0.03	7–9
ΔCt-WT	16.78 ± 1.50	6–7	0.81 ± 0.02	6–10
WT-G312A	17.16 ± 1.00	6–8	0.76 ± 0.04	6–7
G312A-WT	16.84 ± 2.00	6–7	0.79 ± 0.01	6–7
G312A-G312A	7.80 ± 0.50	5–7	0.23 ± 0.02	5–7
G312A-ΔpCt	5.32 ± 0.46	7	0.43 ± 0.03	11–12
G312A-G312A/ΔpCt	3.36 ± 0.37	7–8	0.12 ± 0.05	7–12

*^a^The maximal concentrations of 2-APB used in these curves is 333 μM for these channels, except for ΔpCt-ΔpCt (800 μM). ^b^The number of oocytes used for each point in concentration response curves. ^c^The maximal inhibition produced by pH9.3*.

To further evaluate the gating properties of the concatenated dimer, we also measured its response to extracellular alkalization. When the extracellular pH (pH_o_) was switched from 6.5 to 8.0 or 9.3, the currents of WT-WT channels was drastically inhibited (Figure 1E). Importantly, the dose response curves (Figure 1F) demonstrating that the IRs against their pH_o_ clearly revealed that the tandemly linked dimer was inhibited by extracellular alkalization in a similar degree with monomer. The maximal IR (IR_max_, produced by pH 9.3) for monomeric TREK-2 and WT-WT channels was 0.78 ± 0.02 and 0.73 ± 0.02, respectively (Table 1). Taken together, these data suggest that the tandem-linked strategy does not influence the gating properties of TREK-2 channels. Thus, this method was further used to investigate how the intersubunit and intrasubunit interplay.

### The Proximal C-Terminus Controls the 2-APB Pathway in a Concerted Cooperative Manner Between Subunits

**Figure 2:**
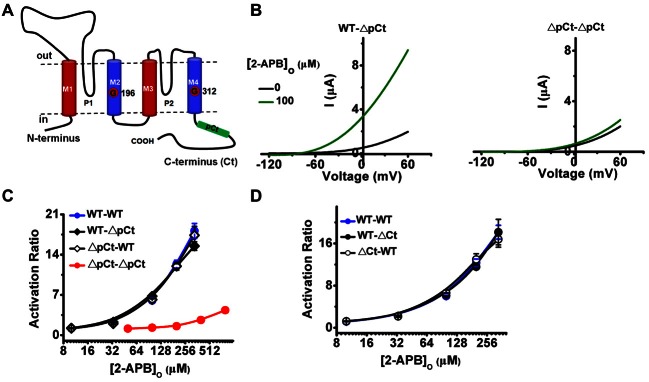
**The effects of incorporation of ΔpCt or ΔCt on the gating processes induced by 2-APB. (A)** Diagram of a single TREK-2 subunit, transmembrane segments 1~4 (M1~M4), N-terminus, the proximal C-terminus (pCt), pore domain 1 (P1) and 2 (P2), glycine hinge of M2 (G196) and M4 (G312) are indicated. **(B)** Exemplar current-voltage recordings from oocytes expressing the indicated channels in the presence of 100 μM 2-APB. **(C,D)** Comparative analysis of the 2-APB evoked activation curves for the indicated channels. Due to the lower sensitivity of ΔpCt-ΔpCt to 2-APB, higher concentrations were used in its curve.

Our previous study have identified that the cytoplasmic pCt (from W326 to A374, the relative position was illustrated in Figure 2A) plays an essential role in gating TREK-2 (Zhuo et al., 2016), so we further used this negative gating mutation to investigate how these two subunits may interact with each other. ΔpCt was constructed as either the first subunit (ΔpCt-WT) or the second subunit (WT-ΔpCt). The response of these heterodimers to 2-APB were investigated and compared with WT-WT and ΔpCt-ΔpCt homodimers.

When the gating of one subunit in TREK-2 dimer does not alter the gating kinetics of another one, the heterodimers should respond to stimuli in a linear combination for their two homodimers (such as WT-WT and ΔpCt-ΔpCt in our study), and such interactive pattern belongs to independent or sequential manner. If the response of heterodimers to stimuli is similar with those of homodimers, the gating between subunits is compatible with concerted cooperative fashion (Zandany et al., 2008; Tombola et al., 2010; Meisel et al., 2012; Thomson et al., 2014). As shown in Figure 2B, 100 μM 2-APB strongly stimulated the currents of WT-ΔpCt, while the activity of ΔpCt-ΔpCt was only slightly stimulated. Comparison of the concentration-dependent curves of the four channels further indicated that the 2-APB responses of both WT-ΔpCt and ΔpCt-WT channels were more similar to that of WT-WT than to that of ΔpCt-ΔpCt (Figure 2C). The AR_max_ for WT-ΔpCt and ΔpCt-WT was 15.50 ± 0.74 and 17.37 ± 1.56, respectively. However, the ratio of ΔpCt-ΔpCt was only 4.34 ± 0.10 even when the concentration of 2-APB was enhanced to 800 μM (Table 1).

Our previous study has also revealed that the full Ct regulates the gating of TREK-2 in a similar manner with pCt (Zhuo et al., 2016), so here we measured 2-APB responses of ΔCt deletant incorporated heterodimers (WT-ΔCt and ΔCt-WT). Consistent with the results from ΔpCt heterodimer, both WT-ΔCt and ΔCt-WT exhibited similar sensitivity to 2-APB as that of WT-WT channels (Figure 2D and Table 1). Taken together, the WT subunit exerts strong, positive effects on the ΔCt or ΔpCt subunit in response to 2-APB stimulation, suggesting that the concerted intersubunit cooperativity gates TREK-2 channels at 2-APB pathway.

### The Proximal C-Terminus Regulates the Extracellular Alkalization-Induced Gating via an Intersubunit Cooperative Manner

**Figure 3:**
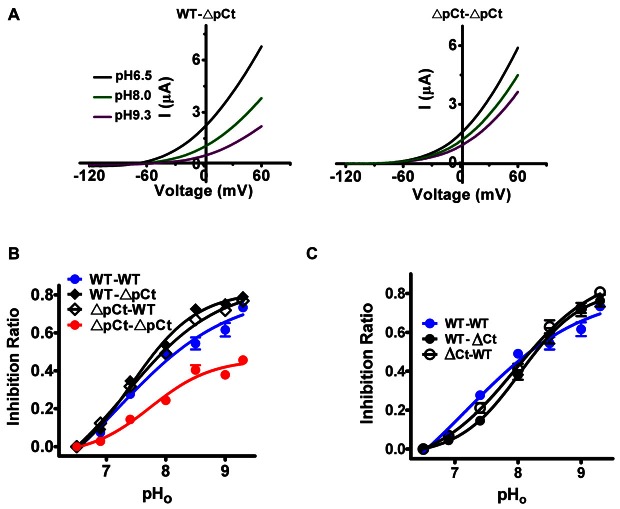
**The effects of incorporation of ΔpCt or ΔCt on the gating processes induced by extracellular alkalization. (A)** Exemplar current-voltage recordings from oocytes expressing the indicated channels as pH_o_ transitions among 6.5, 8.0 and 9.3. **(B,C)** IR of indicated channels to pH_o_ fluctuation.

To confirm above conclusion, we also evaluated the ΔCt- or ΔpC-contained heterodimers to pH_o_ changes (pH_o_ pathway). Similar with the situation of 2-APB response, WT-ΔpCt channels were strongly inhibited by extracellular alkalization, whereas the currents from ΔpCt-ΔpCt channels were slightly blocked (Figure 3A). Furthermore, the responses of WT-ΔpCt (IR_max_ = 0.79 ± 0.01) and ΔpCt-WT (IR_max_ = 0.77 ± 0.02), WT-ΔCt (IR_max_ = 0.76 ± 0.03) and ΔCt-WT (IR_max_ = 0.81 ± 0.02) to pH_o_ changes were more resembled with that of WT-WT channels than that of ΔpCt-ΔpCt channels (Figures 3B,C and Table 1). Taken together, these results reveal that the heterodimers including pCt or Ct deletant have similar sensitivity in response to pH_o_ changes as that of WT-WT channels, suggesting that the pCt (or Ct) domain controls the channel in a concerted intersubunit cooperative manner in the pH_o_ pathway.

### The Glycine Hinge of M4 (G312) also Controls Channel Gating Cooperatively Between Subunits

**Figure 4:**
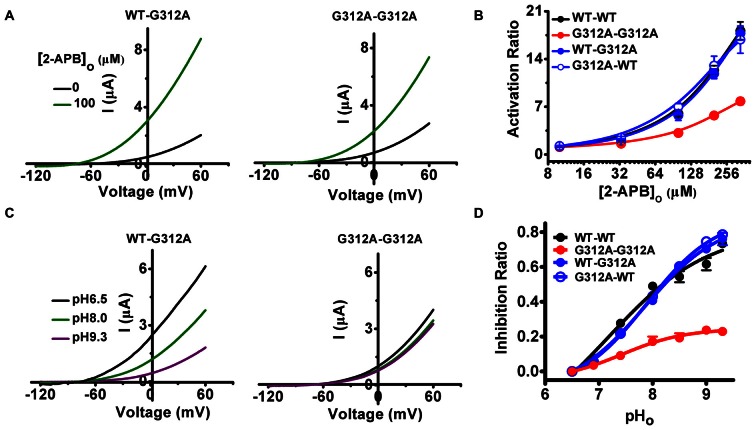
**The effects of incorporation of G312A on the gating processes induced by 2-APB and extracellular alkalization. (A)** Exemplar current-voltage recordings from oocytes expressing the indicated channels in the presence of 100 μM 2-APB. **(B)** Concentration responses of indicated channels activated by 2-APB. **(C)** Exemplar current-voltage recordings from oocytes expressing the indicated channels as pH_o_ transitions among 6.5, 8.0 and 9.3. **(D)** Comparative analysis of pH_o_-inhibition curves for the indicated channels.

The glycine hinge of M4, G312 (the relative position was illustrated in Figure 2A), is involved in the regulations of both 2-APB and extracellular alkalization (Zhuo et al., 2016). By introducing G312A mutation to either the first or second subunit of WT-WT channels and evaluating their responses to 2-APB and ΔpH_o_, we investigated whether G312 gates TREK-2 in a cooperative or seqential manner between subunits. WT-G312A was activated dramatically by 100 μM 2-APB, while the activated degree to G312A-G312A was radically decreased (Figure 4A). The dose response curves of WT-G312A (AR_max_ = 17.16 ± 1.00) and G312A-WT (AR_max_ = 16.84 ± 2.00) were more similar to that of WT-WT channels than to the channel comprising two G312As (AR_max_ = 7.80 ± 0.50; Figure 4B and Table 1). In addition, dimerizing G312A with WT subunit exhibited more resembled sensitivity with WT-WT channels to ΔpH_o_ than that of G312A-G312A (Figures 4C,D and Table 1). These results indicate that G312 of the WT subunit exerts positive effects on the mutated one (G312A) during gating processes induced by 2-APB and pH_o_, suggesting that the glycine hinge of M4 also gates TREK-2 in a concerted cooperative manner between subunits.

### Gly312 and pCt Domain Gate TREK-2 via a cis-Type Mechanism

**Figure 5:**
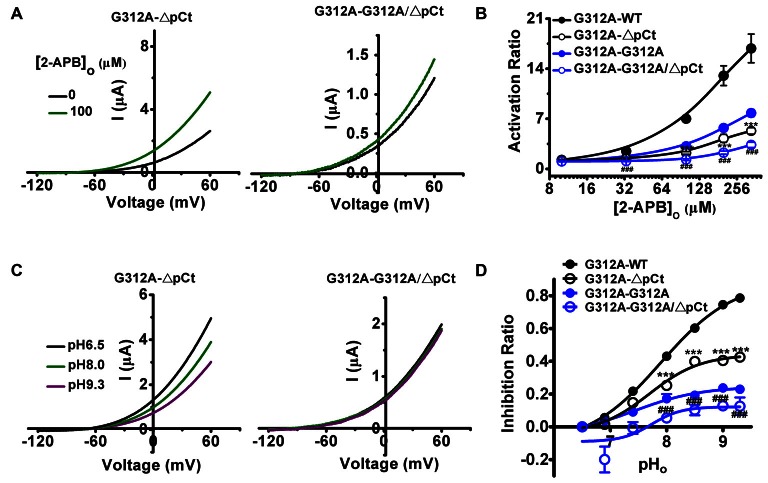
**The effects of further introduction of G312A and ΔpCt, either individually or simultaneously, into the WT subunit of G312A-WT on the gating processes induced by 2-APB and extracellular alkalization. (A)** Exemplar current-voltage recordings from oocytes expressing G312A-ΔpCt and G312A-G312A/ΔpCt channels in the presence of 100 μM 2-APB. **(B)** Concentration responses of indicated channels activated by 2-APB. ***p < 0.001 compared with G312A-WT. ^###^*p* < 0.05 compared with G312A-G312A. **(C)** Exemplar current-voltage recordings from oocytes expressing G312A-ΔpCt and G312A-G312A/ΔpCt channels as pH_o_ transitions among 6.5, 8.0 and 9.3. **(D)** Comparative analysis of pH_o_-inhibition curves for the indicated channels. ***p < 0.001 compared with G312A-WT. ^###^*p* < 0.05 compared with G312A-G312A.

Since both Gly312 and pCt control TREK-2 gating cooperatively between subunits in modulating the bidirectional communication between SF gate and pCt domain (Figures 2–4), it is critical to understand how these elements interact with each other during this communication: a *cis*-type mechanism in which the interaction only occurs within one subunit, or a *tran*s-type mechanism that involves both subunits. To discriminate these two possible mechanisms, we performed mutant cycle analysis. For this purpose, a concatenated dimer with one nonfunctional subunit was required. G312A-WT dimer was chosen because both 2-APB and ΔpH_o_ induced gating pathways were interrupted by G312A mutation in the former subunit. G312A transition and ΔpCt deletion were individually or simultaneously introduced into the WT subunit to obtain G312A-G312A (as also described in Figure 4), G312A-ΔpCt and G312A-G312A/ΔpCt. Then their responses to 2-APB and pH_o_ were investigated. If the interaction between G312 and pCt is *trans*-type, the pCt of the G312A subunit and the G312 of the ΔpCt subunit will rescue the channel function due to their cooperative natures. Accordingly, the effects of 2-APB and pH_o_ changes on G312A-ΔpCt will be similar with those of G312A-WT. Meanwhile, such effects on G312A-G312A/ΔpCt will be analogous with those of G312A-G312A. Nevertheless, the ability of 2-APB to activate G312A-ΔpCt was decreased radically compared with WT-ΔpCt, and such ability was also decreased in G312A/ΔpCt when compared with G312A-G312A (Figure 5A). According to the concentration-dependent curves represented in Figure 5B and Table 1, the AR_max_ of G312A-WT was 16.84 ± 2.00, while the ratio of G312A-ΔpCt was decreased to 5.32 ± 0.46. Likewise, incorporation of ΔpCt into G312A-G312A led to the decrement of AR_max_ from 7.80 ± 0.50 in G312A-G312A to 3.36 ± 0.37 in G312A-G312A/ΔpCt. The pH_o_ response of G312A-ΔpCt also exhibited blunted gating kinetics (Figures 5C,D) when compared with WT-ΔpCt. The IR_max_ of WT-ΔpCt was 0.79 ± 0.01, whereas the ratio of G312A-ΔpCt was decreased to 0.43 ± 0.03 (Table 1). Meanwhile, very similar with the situation in 2-APB, introduction of ΔpCt into G312A-G312A also resulted in blunted pH_o_ response of G312A-G312A/ΔpCt (IR_max_ = 0.12 ± 0.05) when compared with G312A-G312A (IR_max_ = 0.23 ± 0.02; Figures 5C,D). Taken together, further deleting the function of pCt of the WT subunit in G312A-WT or G312A-G312A channel attenuates its sensitivity to 2-APB and pH_o_ changes, suggesting that the allosteric regulations triggered by these stimuli occur along one subunit, rather than across both subunits. Namely, G312 and pCt gate TREK-2 channels via a *cis*-type mechanism.

## Discussion

It has been well established that the activities of K2Ps are tuned by a variety of stimuli from extracellular or intracellular side, nevertheless, how the two subunits respond to these stimuli remains largely unknown. The main purpose of this study was to determine the nature of intersubunit interaction during TREK-2 gating, using concatenated dimeric TREK-2 constructs. By determining the effects of 2-APB and extracellular alkalization on concatenated dimers comprised of WT TREK-2 and loss of function mutants (ΔpCt and G312A), we demonstrate that the two subunits gate channel in a cooperative concerted way, and show that the allosteric regulations evoked by 2-APB and pH_o_ changes occur in a *cis*-type mechanism.

In homooligomeric proteins, allosteric regulation is often achieved by conformational changes, which may lead to cooperativity between subunits. Such intersubunit cooperativity may be concerted (Monod-Wymann-Changeux model; Monod et al., 1965) or sequential (Koshland-Nemethy-Filmer model; Koshland et al., 1966). To identify the nature of intersubunit cooperativity, researchers generally use concatenated oligomers comprising of WT or mutated subunit. If all the subunits contribute to the gating mechanism, which is manifested by different behavior between homooligomers (either WTs or mutants) and heteroligomers, the cooperativity is sequential. For example, KCNQ1 (Kv7) channels experience sequential gating transitions with independent voltage sensor domain movements, in which the relationship between the shift in the voltage dependence of activation and the number of mutated subunits is linearity (Meisel et al., 2012). Otherwise, if the phenotype of heteroligomers is similar with those of homooligomers, either WT or mutated, the cooperativity between subunits belongs to concerted all-or-none manner. To identify the nature of intersubnit interaction in TREK-2, we constructed tandemly linked dimer, and used loss of function mutation (G312A) or deletion (ΔpCt or ΔCt) to mark the function of single subunit. Deletion of pCt domain (or the full Ct) in a single subunit reserved the normal response to 2-APB and pH_o_ changes (Figures 2, 3). In addition, destructing the role of G312 in a single subunit did not affect the sensitivity of TREK-2 to 2-APB and pH_o_ changes (Figure 4). These results clearly indicate that at least the glycine hinge of M4 and the pCt domain gate the channel in a intersubunit concerted cooperative manner in both 2-APB and pH_o_ pathways. Together with our previous study demonstrating that both pCt and G312 are common gating elements used by these bidirectional pathways (Zhuo et al., 2016), the concerted intersubunit interaction during both pathways further supports that similar gating mechanism may be utilized by inter- and extracellular stimuli in regulating the activity of TREK-2.

In our opinion, the concerted cooperativity could further be classified into two manners: positive and negative. If one single mutated subunit is able to eliminate the associated phenotype of WT channel, the concerted cooperativity is negative, the so-called all-or-none mode. Such manner has been found in the activation gate opening of the *shaker* Kv channel (Zandany et al., 2008), the slow deactivation (Thomson et al., 2014), C-type inactivation (Wu et al., 2014a), and drug-binding induced inactivation of human ether-à-go-go-related Gene K^+^ channel 1 (hERG1; Wu et al., 2015b). Otherwise, if one single WT subunit is sufficient to maintain the phenotype of WT channel, the intersubunit interaction is compatible with positive cooperativity, which means the WT subunit exert positive or dominant effects to the mutated ones. The heterodimers comprising of WT and G312A/ΔpCt completely reserve the sensitivity of TREK-2 to 2-APB and ΔpH_o_, suggesting the cooperativity of the two subunits in TREK-2 acts in a positive manner. Such positively concerted cooperativity has only been found in other homomeric channels. In the blocking of α-bungarotoxin to homopentameric α7 acetylcholine receptors, a single subunit confers nearly maximal suppression of channel opening (daCosta et al., 2015). A voltage-gated proton homodimeric channel, Hv1, utilizes the positive mechanism to harness its voltage sensed gating (Tombola et al., 2010). TREK-1, the closest relative of TREK-2 in K2Ps (Lesage et al., 2000), functions normally when dimerized with loss of function deletant (ΔCt) in the presence of either inter- or extracellular stimuli (Sandoz et al., 2012). Single pH_o_ sensor of TASK2, another member of K2Ps, is also sufficient to rescue the pH_o_ sensitivity of the channel (Zúñiga et al., 2011).

Mechanistically, two possibilities might explain the phenotype of heterodimers (combinations of WT and G312A or WT and ΔpCt; cooperative concerted gating properties) in TREK-2: first, the movements of WT subunit might lead to similar movements of the mutated or deleted subunit, so that the phenotype of heterodimer is analogous to those of WT-WT. Second, the movements of WT subunit are sufficient to manipulate the pore of TREK-2 to control the efflux of potassium ions. Although the negative gating subunits are not able to control gating, they might be necessary to maintain the normal structure of the pore. That is, they might exert structural function. Although we tend to believe that the second possibility rarely occurs, our data and current crystallographic evidence are not capable of excluding it completely.

Although both G312 and pCt domain gate TREK-2 in a intersubunit cooperative fashion in both 2-APB and pH_o_ pathways, the nature of such cooperativity might depend on the specific mutation or stimuli used to the gating process. A recent study reveal that both sensors of ruthenium red (D135), located on the extracellular side, contribute to its inhibitory effect on TREK-2 (Braun et al., 2015), implicating that TREK-2 might also adopt intersubunit sequential or independent mechanism to gate the pore. Interestingly, although one Ct is sufficient to gate the pore in the regulations by extracellular acidification and G protein coupled receptors in TREK-1 (Sandoz et al., 2012), which suggests positive cooperativity, it seems that Ct also gates the pore via a negatively cooperative mechanism, as demonstrated by other group (Bagriantsev et al., 2012). Thus, data related to the gating mechanisms of channel with concatenated heteromeric channels should be carefully interpreted, as that suggested in the C-type inactivation and drug binding studies of hERG1 channels (Wu et al., 2014a, 2015a,b).

Since the movements of M4 and pCt domain gate the pore in both 2-APB and pH_o_ pathways in a completely concerted cooperative manner, we further determined whether *cis*- or *trans*-type mechanism mediates G312 and pCt in the crosstalk between SF gate and cytosolic pCt domain in TREK-2. According to our results, further introduction of ΔpCt into the latter subunit of G312A-WT and G312A-G312A blunted their responses to both 2-APB and ΔpH_o_ (Figure 5), suggesting that the bidirectional signal transduction between pCt domain and SF gate via M4 is not able to occur across two subunits. Namely, they gate TREK-2 through a *cis*-type mechanism, with transduction along one single subunit. Similar mechanism has also been found in TREK-1 when regulating temperature response (Bagriantsev et al., 2012).

Mechanistically, the energy gained from occupancy by a single 2-APB or alkalization of a single pH_o_ sensor might be sufficient to manipulate channel gating, and allow the signals be transduced along the subunit in TREK-2. A possible interpretation is that, another subunit might only exert structural functions at least in these stimuli-induced gating, implicating the function of the channel is not associated with its dimeric status. Consistently, several studies have reported that some K2Ps do not require the disulfide bond for formatting homodimer or their functions (Patel et al., 2000; Niemeyer et al., 2003; Hwang et al., 2014).

In summary, our data suggest that the WT subunit exerts fully dominant roles to the mutated one by tagging one of the subunit types with conductance mutations to report subunit stoichiometry. And further incorporating conductance mutations into the heterodimeric channels suppress both pathways. These results support that intersubunit concerted cooperative and *cis*-type mechanism control the pore gating of TREK-2 during 2-APB and ΔpH_o_-induced transductions.

## Author Contributions

X-YM, R-GZ, and X-LW conceived the study, engineered mutant constructs, performed electrophysiological experiments, collected and analyzed data, and wrote the manuscript. PP and X-YL performed electrophysiological experiments. H-TY constructed mutant channels. J-PX and J-QZ edited the manuscript.

## Conflict of Interest Statement

The authors declare that the research was conducted in the absence of any commercial or financial relationships that could be construed as a potential conflict of interest.
